# Supplementary figures and images for: Beyond RuBisCO: convergent molecular evolution of multiple chloroplast genes in C4 plants
Source: PeerJ. 2022 Jan 27;10:e12791. doi: 10.7717/peerj.12791 (PMC8801178; doi:10.7717/peerj.12791)

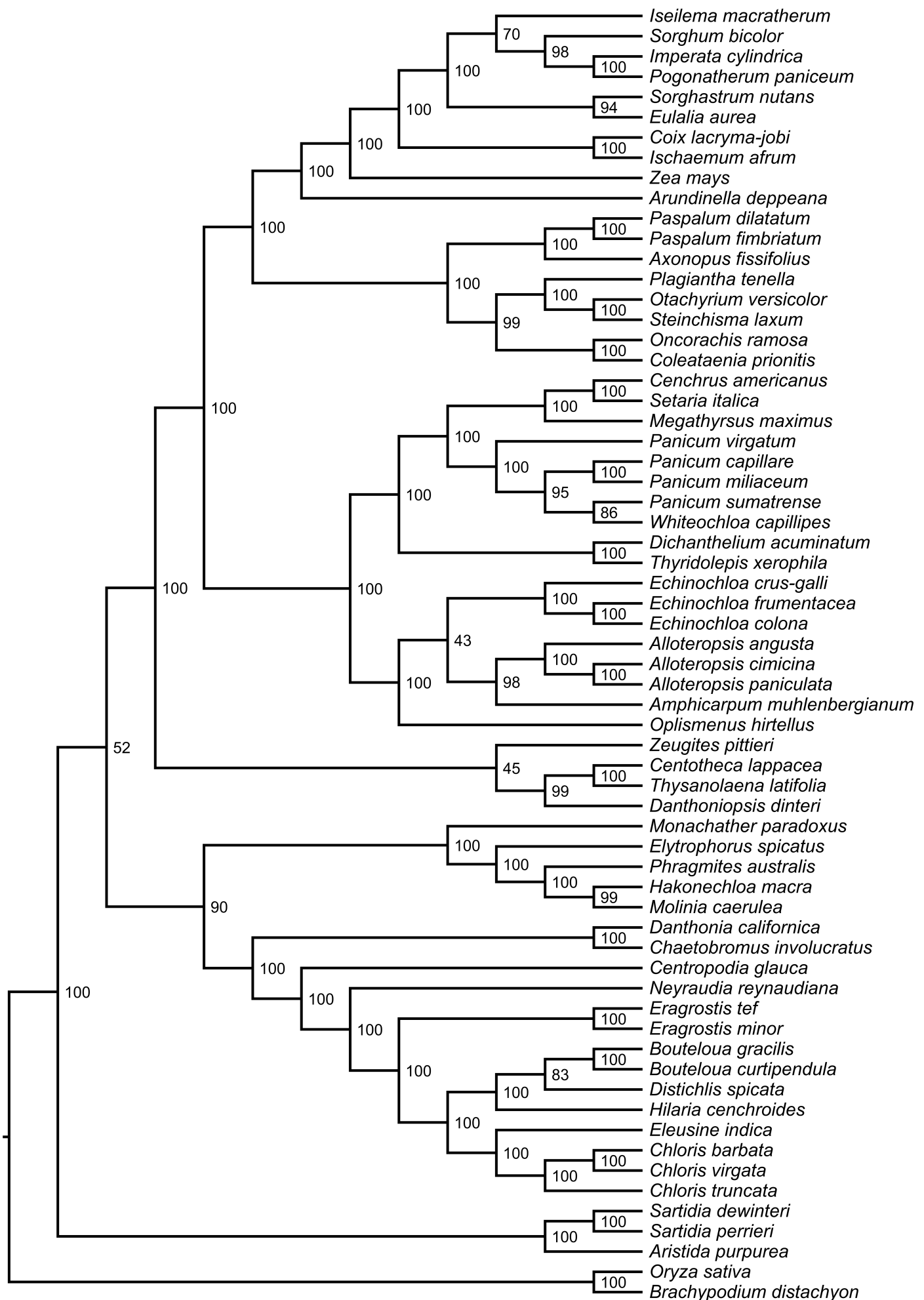

Supplement: Supplemental Information 8 — The model GTR+ Γ was used. Partitioning scheme was selected using Bayesian Information Criterion (BIC). Numbers represent bootstrap support. [file peerj-10-12791-s008.pdf]

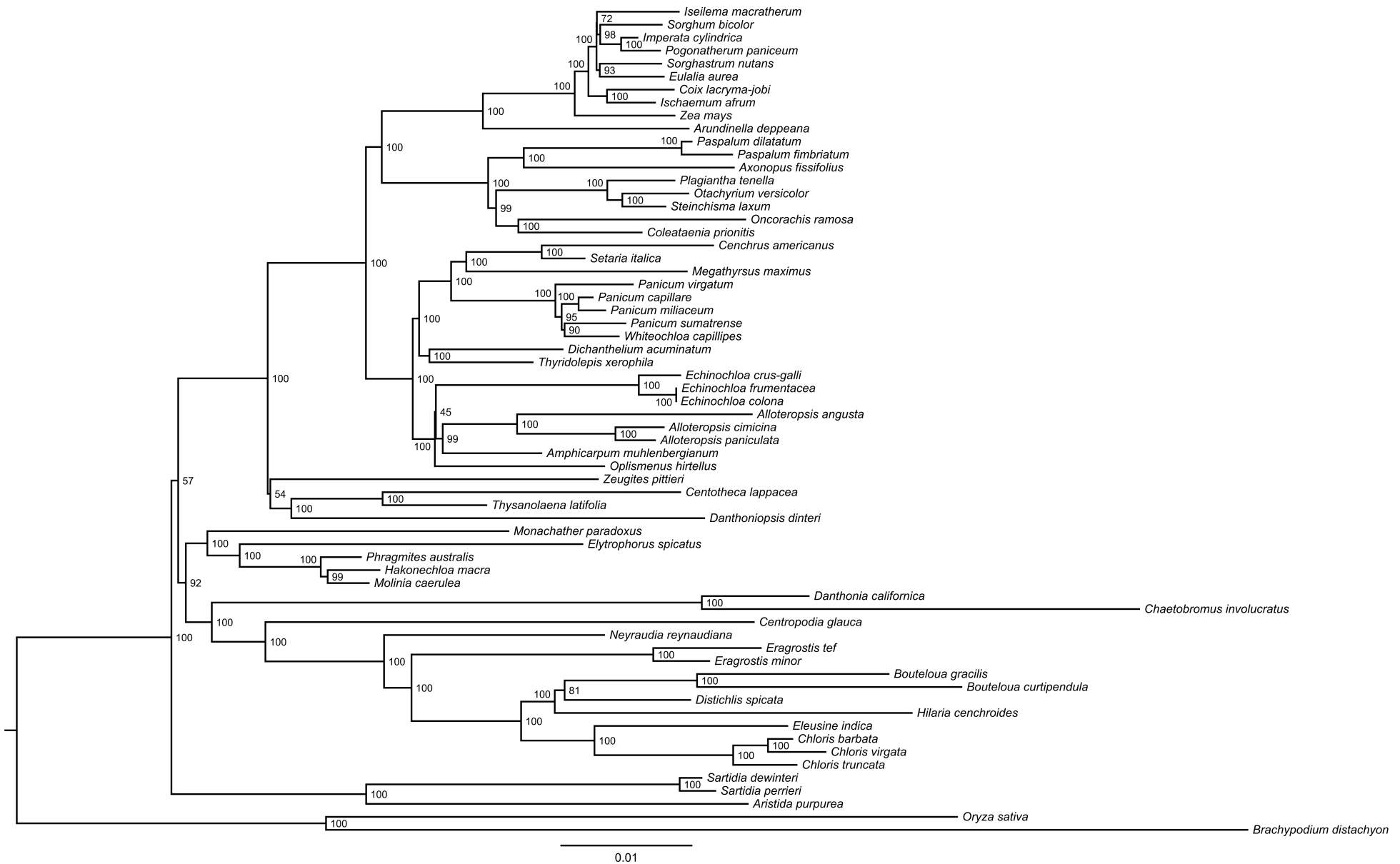

Supplement: Supplemental Information 9 — The model GTR+ Γ was used. Partitioning scheme was selected using Akaike information criterion (AIC). Numbers represent bootstrap support. [file peerj-10-12791-s009.pdf]

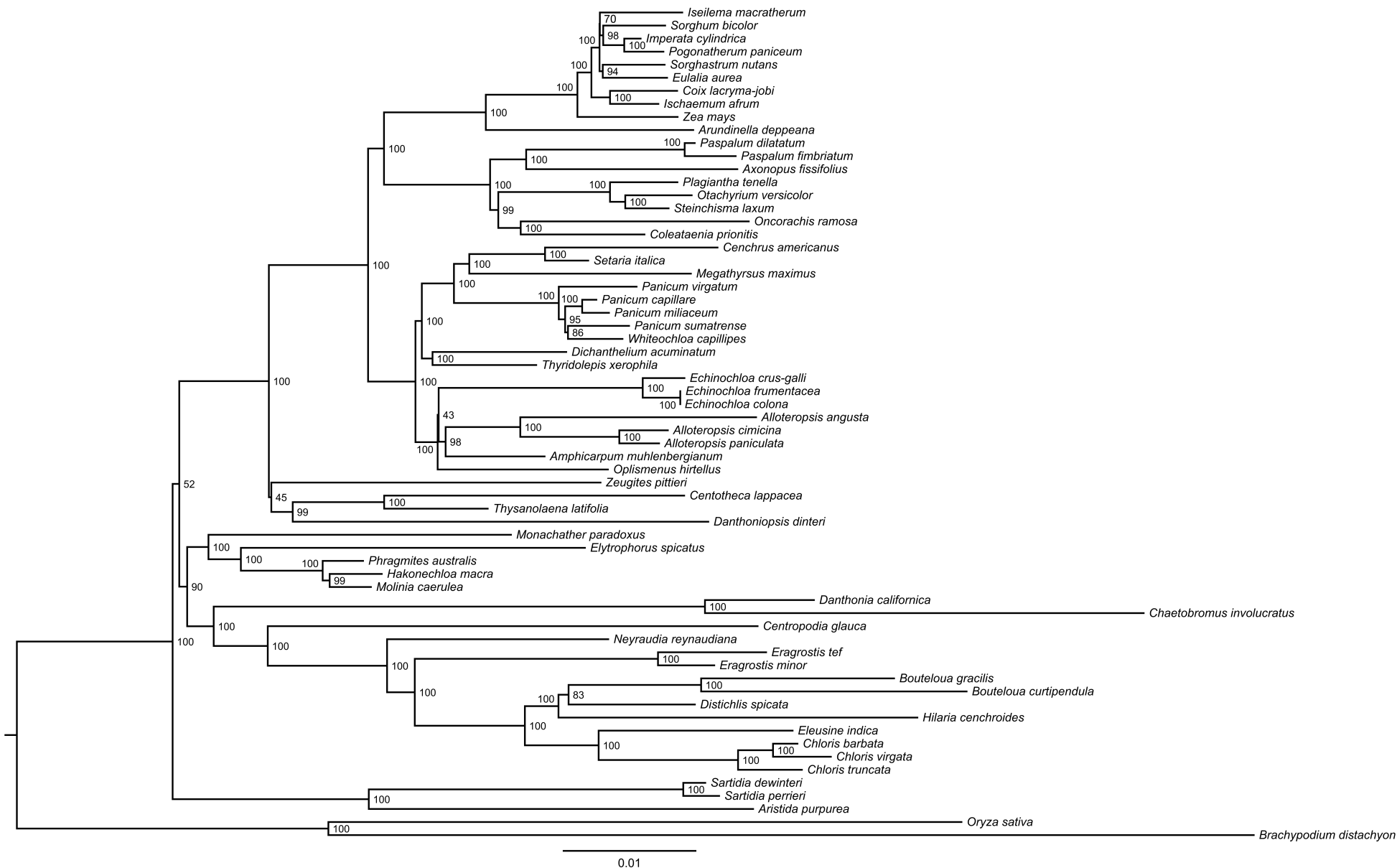

Supplement: Supplemental Information 10 — The model GTR+ Γ was used. Partitioning scheme was selected using Bayesian Information Criterion (BIC). Numbers represent bootstrap support. [file peerj-10-12791-s010.pdf]
